# Elevated FAM134B expression induces radiation-sensitive in hepatocellular carcinoma

**DOI:** 10.1186/s12885-023-11030-x

**Published:** 2023-07-17

**Authors:** Binhui Xie, Yuankang Xie, Cuifu Fang, Baiyin Zhong, Rong Ye, Jianhong Zhang, Qingquan Liu, Heping Li

**Affiliations:** 1 Department of Hepatobiliary Surgery, the First Affiliated Hospital of Gannan Medical University, 341000 Ganzhou, P R China; 2 Department of general surgery III, the First Affiliated Hospital of Gannan Medical University, 341000 Ganzhou, P R China; 3 Department of Medical Oncology, the First Affiliated Hospital of Sun Yat-sen University, 510080 Guangzhou, P R China

**Keywords:** FAM134B, FLT3, HCC, JAK/Stat3 signaling pathway, Radiotherapy-resistance

## Abstract

**Background:**

Previous studies have shown that Family with sequence similarity 134 member B (FAM134B) was involved in the occurrence and development of malignancy, however, the function and molecular mechanism of FAM134B in Hepatocellular Carcinoma (HCC) radiotherapy resistance remain unclear. Therefore, it may clinical effective to clarify the molecular mechanism and identify novel biomarker to overcome radiotherapy resistance in HCC.

**Methods:**

The protein and mRNA expression of FAM134B were determined using Real-time PCR and Western blot, respectively. IHC assay was performed to investigate the association between FAM134B expression and the clinicopathological characteristics of 132 HCC patients. Functional assays, such as in situ model, colon formation, FACS, and Tunel assay were used to determine the oncogenic role of FAM134B in human HCC progression. Furthermore, western blotting and luciferase assay were used to determine the mechanism of FAM134B promotes radiation-sensitive in HCC cells.

**Results:**

We noted that FAM134B was downregulated in HCC, which was correlated with the radiation resistance in patients with HCC. Overexpression of FAM134B contribute to radiation sensitive in HCC; however, inhibition of FAM134B confers HCC cell lines to radiation resistance both in vitro and in vivo. Moreover, we found that FAM134B interacts with FMS related receptor tyrosine kinase 3 (FLT3) and downregulation of FAM134B activated JAK/Stat3 signaling pathway. Importantly, pharmacological inhibition of JAK/Stat3 signaling pathway significantly counteracted downregulation of FAM134B-induced radiation resistance and enhanced radiation therapeutic efficacy in HCC.

**Conclusions:**

Our findings suggest that FAM134B may be a potential therapeutic biomarker for the treatment of HCC patients with radiotherapy tolerance.

**Supplementary Information:**

The online version contains supplementary material available at 10.1186/s12885-023-11030-x.

## Introduction

Liver cancer is one of the malignant tumors with the highest mortality in the worldwide [1, 2]. Hepatocellular carcinoma (HCC) accounts for the majority of primary liver cancer and the outcome for HCC patients were substantially poor, which with a 5 years’ survival rates 10–20% [3, 4]. It has been reported that radiotherapy has the advantages of effectively inhibiting HCC and shooting intrahepatic micrometastasis and play important role in the prevention and treatment of recurrence of HCC [5, 6]. However, due to the liver injury caused by radiotherapy and the low tolerance of the whole liver to radiation, whole liver radiation irradiation is still a scheme that needs to be carefully selected [7]. Although local stereotactic technology can carry out high-dose radiotherapy for the main tumor of liver cancer, it cannot effectively kill the micrometastasis in the liver, so as to prevent the metastasis and recurrence of liver cancer [8]. Therefore, it is of great scientific significance to deeply explore the molecular mechanism of liver cancer cells regulating radiotherapy sensitivity, predict the radiotherapy resistance of liver cancer and improve the radiotherapy efficacy of liver cancer patients.

Regulation of radiotherapy sensitivity is a complex process which included DNA damage repair, anti-radiation of tumor stems cells, regulation of anti-apoptosis ability and regulation of cell cycle arrest. For example, it has been shown that the overexpression of ATP binding cassette transporter protein G2 (ABCG2) in tumor stem cells significantly enhance the natural ability to resist radiation, resulting in tumor radiotherapy tolerance [9]. Furthermore, the classical apoptosis related genes p53, caspase, Mcl-1 and PTEN have been proved to be closely related to the formation of radiotherapy resistance of cancer [10]. In addition, CXCR4, a tumor stem cell related gene closely related to the occurrence and development of liver cancer, has been proved to affect the sensitivity of radiotherapy in a variety of cancers [11]. Moreover, other studied about hypoxic microenvironment, autophagy and angiogenesis are also involved in regulating the radiosensitivity of tumor cells [12, 13]. Although there are some studies on the regulation mechanism of radiotherapy sensitivity of liver cancer, it is not comprehensive. Therefore, it is of great scientific significance to deeply explore the molecular mechanism of liver cancer cells regulating radiotherapy sensitivity, predict the radiotherapy sensitivity and resistance of liver cancer and improve the radiotherapy efficacy of liver cancer patients.

Family with sequence similarity 134-member B (FAM134B) was firstly found in esophageal squamous cell carcinoma and encodes a 497 amino acid CIS Golgi transmembrane endoplasmic reticulum receptor protein, which regulates the turnover of endoplasmic reticulum through selective phagocytosis and affects the endoplasmic reticulum stress of cells [14–16]. It is well known that endoplasmic reticulum stress can make cancer cells (such as esophageal squamous cell carcinoma) adapt to the tumor microenvironment and promote cancer growth [17, 18]. Meanwhile, endoplasmic reticulum stress can also induce apoptosis of cancer cells (such as colon cancer cells and breast cancer cells) by inducing p53 [19]. Recent reports indicate that FAM134B is highly expressed in hepatocellular carcinoma and enhances the biological functions of hepatocellular carcinoma cells such as proliferation, invasion and metastasis by regulating AKT signaling pathway [20]. Oliver P Forman and colleagues reported that FAM134B was identified to associate with sensory neuropathy in the border collie dog breed by RNAseq experiments [21]. Furthermore, mutations of FAM134B were also found to be association with ESCC malignant progression [16]. However, the biological function and molecular mechanism of FAM134B in radiotherapy sensitivity of HCC are still unclear, it would be worthy to enunciate the biological effects and molecular mechanisms of FAM134B in radiotherapy sensitivity.

## Materials and methods

### Cell culture

The HCC cell lines used in this study were purchased from the American Type Culture Collection (ATCC, Manassas, VA, USA) and were grown in Dulbecco’s modified Eagle’s medium (add with 10% fetal bovine serum) and cultured in a humidified incubator (37 °C, 5% CO2 atmosphere).

### Tissue specimens

Our study included 132 patients diagnosed as HCC. The details of clinical tumor tissues are listed in the Tables 1 and 2. Prior patient consent and approval were obtained from the Institutional Research Ethics Committee. The number details of ethics approval are 2,020,064. Inclusion criteria of the tissue samples in our study was: patients who have been diagnosed as HCC by pathology and have received radiotherapy can be the research object of this project. By querying the patient’s medical record, the patient’s past clinical tumor tissue can be traced. Exclusion criteria: patients with HCC who cannot trace to clinical tumor tissue and patients with HCC who have not received radiotherapy are excluded from the project.

**Table 1:** Clinicopathological Characteristics of Studied Patients and Expression of FAM134B in HCC

Characteristics	Number of cases
**Gender**	
Male	118
Female	14
**Age(years)**	
> 45	78
≤ 45	54
**Clinical Stage**	
I	88
II	31
III	9
IV	4
**T classification**	
T1	71
T2	23
T3	7
T4	31
**N classification**	
N0	121
N1	11
**M classification**	
Yes	4
No	128
**Cirrhosis**	
Yes	67
No	65
**HBsAg**	
Yes	115
No	17
**HCV**	
Yes	2
No	130
**FAM134B**	
High expression	65
Low expression	67
**Survive or Mortality**	
Survive	22
Mortality	110

**Table 2:** Univariate and multivariate analyses of various prognotic parameters in patients with Liver Cancer by Cox-regression analysis

	Univariate analysis			Multivariate analysis		
	No. patients	*P*	Relative risk	*P*	Relative risk	95% confidence interval
Expression of FAM134B						
Low expression	67	0.002	0.555	0.036	0.646	0.429–0.973
High expression	65					
Clinical Stage						
I & II	119	0.001	1.464	0.036	1.320	1.018–1.711
III & IV	13					

### Plasmid construction and transfection

Human FAM134B coding sequence was subcloning into pMSCV vector (Clontech, Mountain View, CA) to generate FAM134B overexpressing plasmid. Two siRNA oligonucleotides were cloned to generate pSuper-retro-shFAM134B#1, shFAM134B#2, respectively. In this study, Lipofectamine 3000 reagent (Invitrogen, Carlsbad, CA) was used for cell transfection and stable cell lines were selected for 10 days with 0.5 µg/ml puromycin 48 h after infection.

### Western blotting

Western blotting was performed to analyze the protein level of FAM134B, p-JAK2 and p-STAT3 in our study. Anti-FAM134B antibody (Cell Signaling, Danvers, MA, USA, 1:500), anti-p-JAK2(1:500), anti-JAK2(1:500), anti-p-STAT3(1:1000), anti-STAT3(1:500) (Cell Signaling, Danvers, MA, USA). The membranes were then stripped and re-probed with an anti- β-actin monoclonal antibody (Cell Signaling, 1:3000) as a loading control.

### Xenografted tumor model

24 BALB/c-nude mice (5–6 weeks of age, 18-20 g) were used in our study and purchased from the Center of Experimental Animal of Guangzhou University of Chinese Medicine. The BALB/c nude mice were randomly divided into two groups (*n =* 6/group). Mice was inoculated with Hep3B /Vector cells (5 × 10^6^) or with Hep3B/ FAM134B cells (1 × 10^6^) by using the orthotropic model. Tumors were examined once ten days by an IVIS imaging system. On day 50, tumors were collected, and AST and ALT indexes were detected. All experimental procedures were approved by the Institutional Animal Care and Use Committee.

### Luciferase assay

Luciferase assay was performed to analyze the Stat3 luciferase levels. 100ng of pGL3-luciferase plasmid was transfected into HCC cells using the Lipofectamine 3000 reagent. Luciferase and control signals were measured at 48 h after transfection using the Dual Luciferase Reporter Assay Kit (Promega), according to a protocol provided by the manufacturer. Three independent experiments were performed, and the data were presented as the mean ± SD.

### Immunohistochemistry (IHC)

Immunohistochemistry (IHC) analysis was performed on the 132 paraffin-embedded HCC tissue sections as previously described. The IHC staining results were assigned a mean score and were reviewed and scored separately by two independent pathologists. The intensity was scored as follows: 1, no staining; 2, weak staining (light yellow); 3, moderate staining (yellow brown); 4, strong staining (brown). Tumor cell proportions were scored as: 0, no positive tumor cells; 1, < 10% positive tumor cells; 2, 10–35% positive tumor cells; 3, 35–75% positive tumor cells; 4, > 75% positive tumor cells. The staining index (SI) was calculated as the product of the staining intensity score and the proportion of positive tumor cells (SI: 0, 2, 3, 4, 6, 8, 9, 12, and 16). Samples with a SI ≥ 8 were determined as high expression and samples with a SI < 8 were determined as low expression. Cutoff values were determined on the basis of a measure of heterogeneity using the log-rank test with respect to overall survival.

### Annexin V assay

Annexin V assay was performed to analyze the anti-apoptotic effect of FAM134B. PE Annexin V Apoptosis Detection Kit I (BD Pharmingen) was used and all experimental steps shall be carried out according to the experimental instructions. The percentage of apoptosis was analyzed with an EPICS XL flow cytometer (Beckman-Coulter). Each sample was analyzed in triplicate.

### Colony formation assays

1000 HCC cells were plated on 6 well plates and cultured for 10 days. The colonies were stained with 1.0% crystal violet for 30s after fixation with 10% formaldehyde for 5 min.

### Statistical analysis

The statistical methods used in this subject are as follows: Cox regression model, Fisher’s exact test, log-rank test, Chi-square test, and Student’s 2-tailed t test, which were performed using the SPSS 21.0 statistical software package. Data represent mean ± SD. *P* < 0.05 was considered statistically significant.

## Results

### FAM134B is downregulation and correlates with radiation sensitive in HCC

**Fig. 1:**
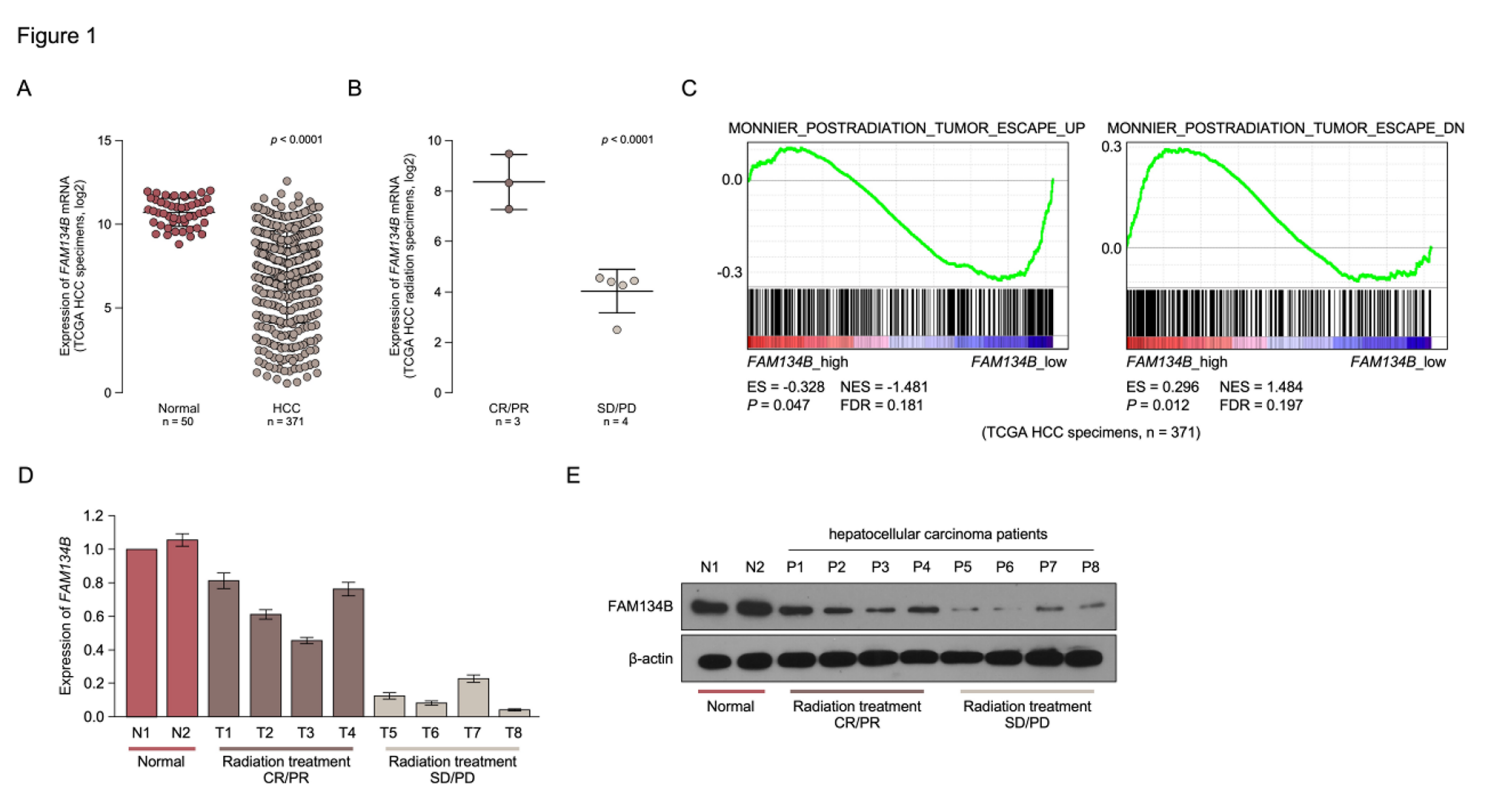
**FAM134B is downregulation and correlates with radiation sensitive in HCC. A.** Downregulated FAM134B in tumor tissue compared with normal tissue (n = 421, TCGA). **B**. FAM134B was downregulated in HCC who revised radiotherapy and exhibit stable disease (SD) or progressive disease (PD), compared with patient’s exhibit (PD) complete response (CR) or partial response (PR) (n = 7, TCGA). **C** GSEA plot analyze the correlation between the mRNA levels of FAM134B and post-radiation gene signatures in published datasets. **D** Real-time PCR analysis showed FAM134B expression was decrease in 8 HCC tissues. **E** Western blotting analysis showed FAM134B expression was decrease in 8 HCC tissues. β-actin was used as a loading control

By analyzing a published microarray-based high-throughput assessment, FAM134B was identified to be significantly downregulated in multiple tumor (Supplemental Fig. 1A) and HCC tissues compared with non-tumor tissue (Fig. 1A, TCAG- hepatocellular carcinoma, n = 421, *P* < 0.001; Supplemental Fig. 1B, E-GEOD-14,520, n = 445; *P* < 0.001). Interestingly, FAM134B was identified to be significantly downregulated in HCC who revised radiotherapy and exhibit stable disease (SD) or progressive disease (PD), compared with patient’s exhibit (PD) complete response (CR) or partial response (PR) (n = 7; *P* < 0.001; TCAG- hepatocellular carcinoma; Fig. 1B). Furthermore, FAM134B overexpression was strongly converse correlated with with postradiation in TCGA dataset of HCC by performing GSEA analysis (Fig. 1C). Real-time PCR analyses and western blot revealed that FAM134B was decrease in all 8 hepatocellular carcinoma cell lines at both the protein and mRNA levels, compared with non-tumor tissue (Fig. 1D and E). Importantly, FAM134B was markedly decrease in HCC patients who revised radiotherapy and exhibit SD or PD compared with patient’s exhibit CR or PR. These results suggest that FAM134B is downregulation and correlates with radiation sensitive in HCC.

### FAM134B downregulation correlates with progression and poor prognosis in HCC patients

**Fig. 2:**
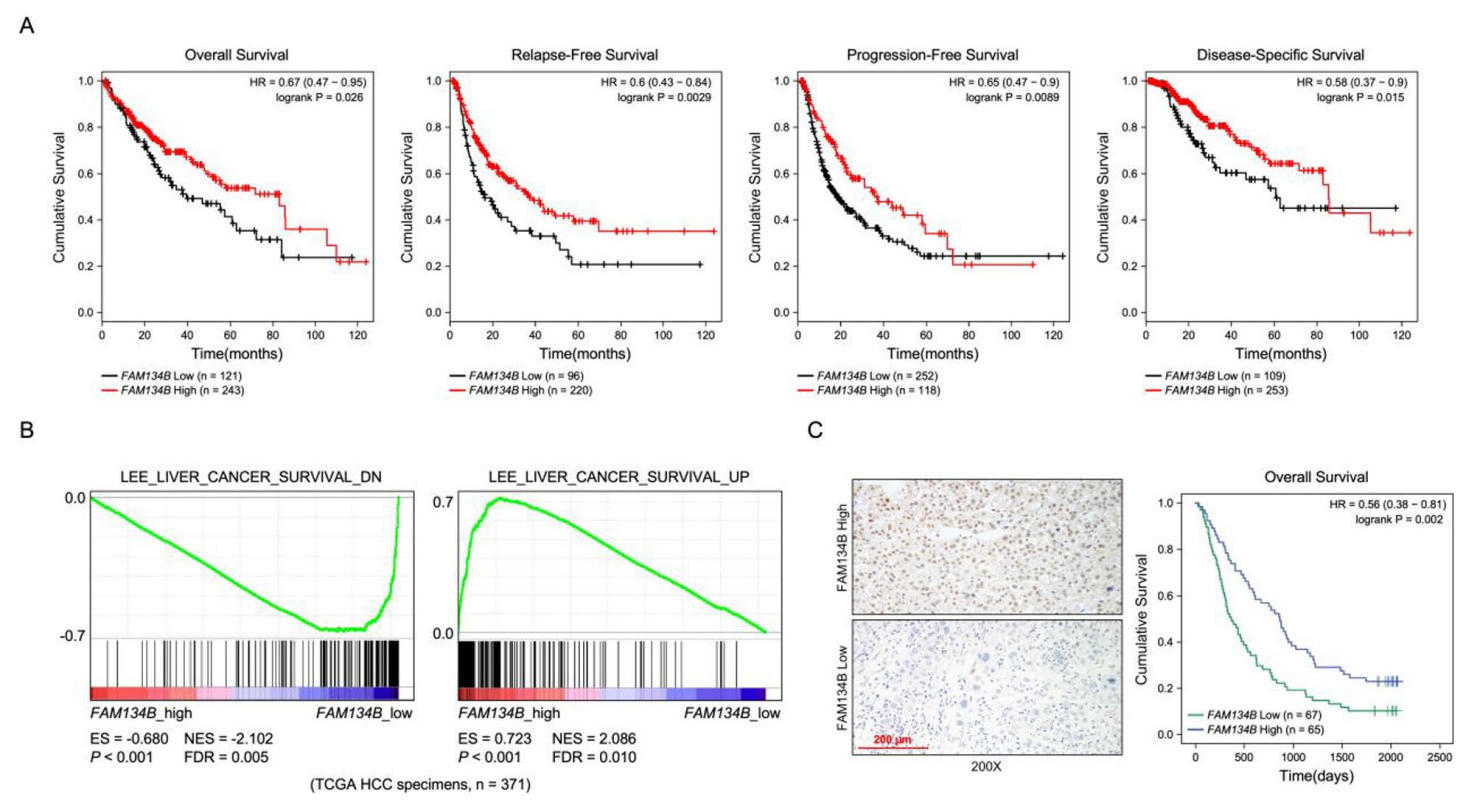
**FAM134B downregulation correlates with progression and poor prognosis in HCC patients** **A** Survival analysis of FAM134B expression in HCC patients. **B** GSEA plot analyze the correlation between the mRNA levels of FAM134B and the liver cancer survival in published datasets. **C** IHC staining analyze the FAM134B protein expression in HCC (left); The Kaplan-Meier survival curves compare HCC patients with low and high FAM134B expression levels (right), *P* < 0.001

Moreover, Kaplan-Meier plotter analysis http://kmplot.com/analysis/index.php?p=service&cancer=liver_rnaseq. revealed that hepatocellular carcinoma patients with lower FAM134B expression was association with overall survival (p = 0.026), relapse-free survival(p = 0.0029), progression-free survival(p = 0.0089) and disease-specific survival(p = 0.015) (Fig. 2A). GSEA revealed that FAM134B decrease strongly correlated with liver cancer survival in TCGA dataset of HCC (Fig. 2B). Survival analysis showed that lower FAM134B expression had significantly worse overall than those with higher FAM134B expression in HCC (Fig. 2C). Moreover, univariate and multivariate Cox regression analysis revealed that Clinical Stage (HR = 1.320, 95% CI = 1.018–1.711, *P* = 0.036) and FAM134B expression (HR = 0.646, 95% CI = 0.429–0.973, *P* = 0.036) were each recognized as independent prognostic factors in HCC (Table 2). Collectively, these results suggest a positive correlation between FAM134B expression and HCC progression.

### FAM134B contribute to radiation sensitive in HCC in vitro

**Fig. 3:**
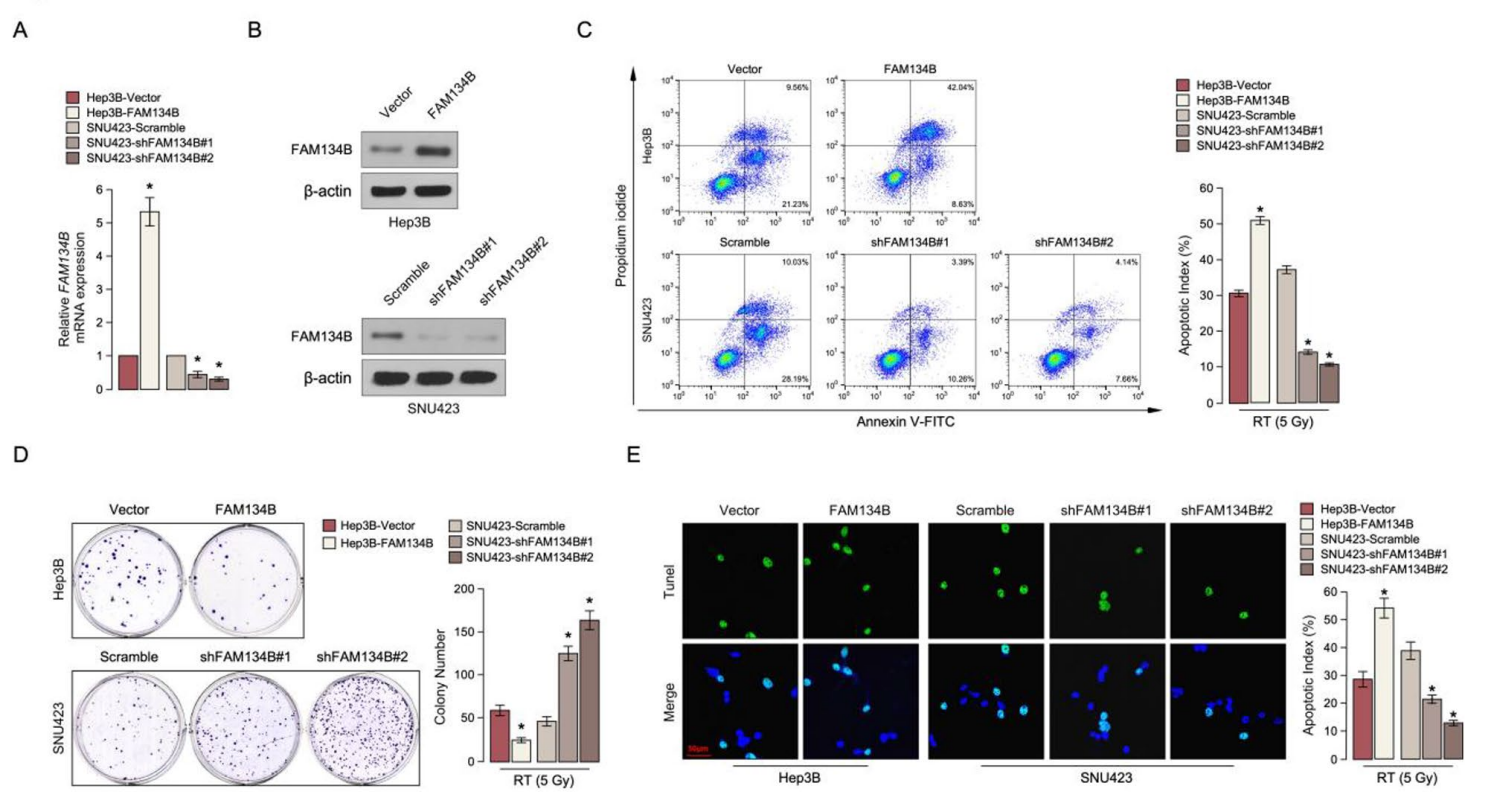
**FAM134B contribute to radiation sensitive in HCC** ***in vitro*** **A.** Real-time PCR analysis of FAM134B expression in the indicated cells. **B.** Western blotting analysis of FAM134B expression in the indicated cells. β-actin was used as a loading control. **C.** Annexin V-FITC and PI staining of the indicated cells. **D.** Representative micrographs (left) and quantification (right) of colonies in the colony formatiom assay. **E.** Representative micrographs (left) and quantification (right) of Tunel positive signaling in the indicated assay. Each bar represents the mean ± SD of three independent experiments. * *P* < 0.05

To investigate the radiation sensitive role of FAM134B in HCC progression, the protein level of FAM134B was firstly examined in HCC cell lines (Supplemental Fig. 2A-2B). Hep3B cell line which shown lower FAM134B expression was stably overexpress FAM134B, and SNU423 which shown higher FAM134B expression was stably inhibit FAM134B were established (Fig. 3A and B). Annexin V and Tunel staining assay show that the percentage of apoptotic cells in SNU423/shFAM134B HCC cells treated with radiation (5 Gy) was much lower compared than that in control cells, but much higher in Hep3B/ FAM134B cancer cells (Fig. 3C and E). Strikingly, colony formation assay shows that overexpression of FAM134B demonstrates lower growth rates of HCC cells treated with radiation (5 Gy) compared to vector-control cells, but knockdown FAM134B have the opposite effect (Fig. 3D). The above results indicating that deregulation of FAM134B is involved in radiation sensitive of HCC cells.

### FAM134B contribute to radiation sensitive in HCC in vivo

**Fig. 4:**
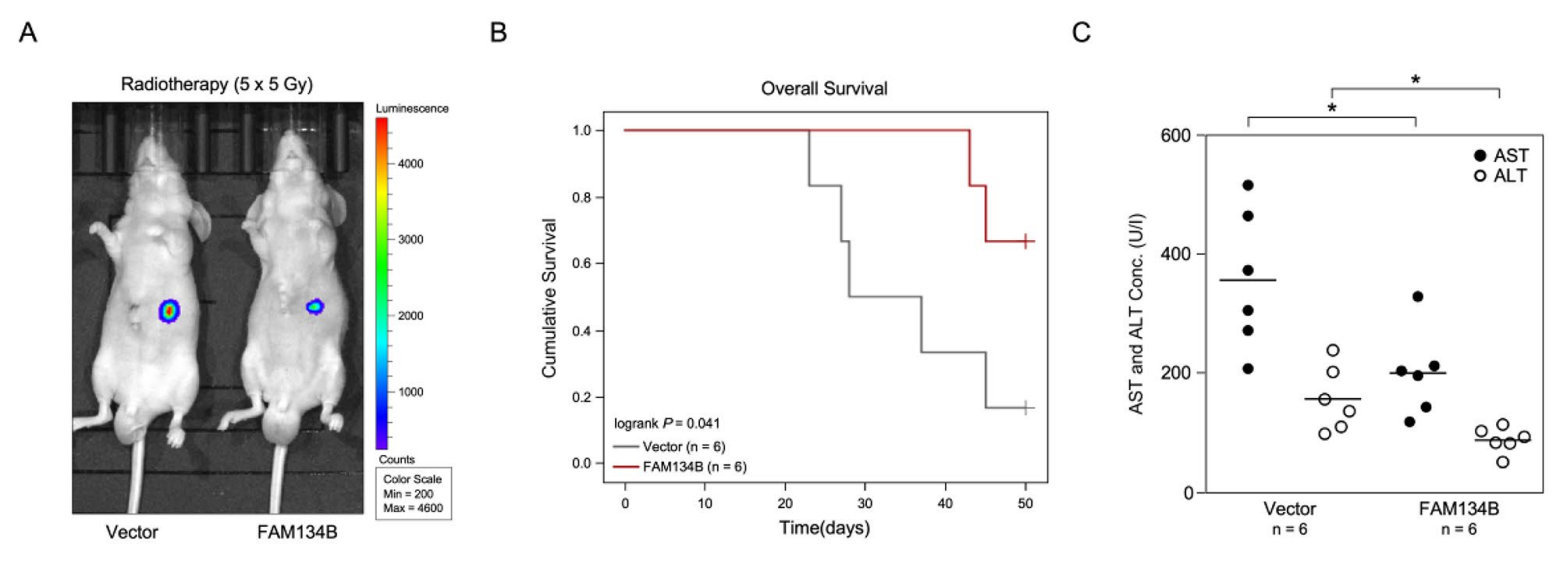
**FAM134B contribute to radiation sensitive in HCC** ***in vivo*** **A.** The luminescence of the tumor xenografts in the indicated groups. **B.** The Kaplan-Meier survival curves compare mice with low and high FAM134B expression levels. **C.** Concentration of AST and ALT in the indicated groups. * *P* < 0.05

The capability of FAM134B in radiation sensitive of HCC was further examined using an in vivo in situ model with radiation treatment (5 × 5 Gy). In agreement with the in vitro results, FAM134B-overexpression cells showedsmaller tumors than the control cells (Fig. 4A) and mice with high FAM134B expression showed better overall survival compared to vector-control cells (Fig. 4B, P = 0.041). Importantly, the AST and ALT levels was significantly lower in FAM134B overexpression group (Fig. 4 C).

### Downregulation of FAM134B activates the JAK/Stat3 signaling pathway in HCC

**Fig. 5:**
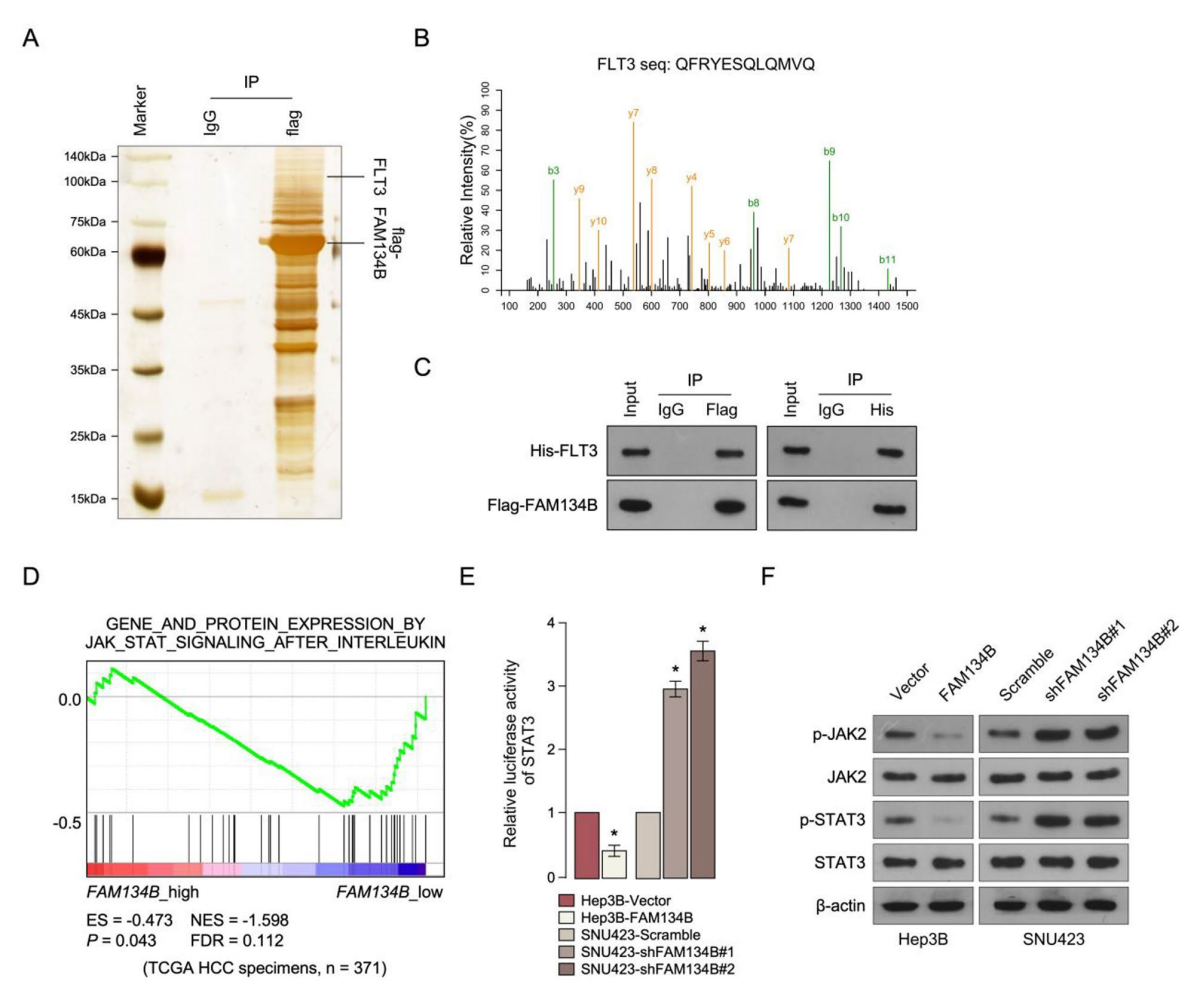
**Downregulation of FAM134B activates the JAK/Stat3 signaling pathway in HCC.** **A.** Coomassie brilliant blue analysis of proteins interacting with FAM134B. **B.** MS sequencing showing that FAM134B interacted with FLT3. **C.** Co-IP assay showing that FAM134B interacted with FLT3. **D.** GSEA plot analyze the correlation between the mRNA levels of FAM134B expression in HCC published datasets. **E.** Analysis of luciferase reporter activity in the indicated cells. **F.** Western blotting analysis of the expression levels of indicate proteins in the indicated cells. β-actin was used as a loading control. Each bar represents the mean ± SD of three independent experiments. * *P* < 0.05

In order to better understand the mechanism of downregulation of FAM134B induced radiation-resistance, the potent proteins which interaction with FAM134B was identified by immunoprecipitation/mass spectrometry (IP/MS) (Fig. 5A). IP/MS and co-IP analyses demonstrated that FAM134B interacts with FMS related receptor tyrosine kinase 3 (FLT3) (Fig. 5B C). Further GSEA analysis was performed and shown that FAM134B mRNA expression levels were conversely correlated with JAK/Stat3 signaling pathway gene signatures (Fig. 5D). Furthermore, FAM134B significantly reduced, but inhibition of FAM134B enhanced Stat3 luciferase reporter activity in HCC cells (Fig. 5E). FAM134B-overexpressing cells showed decrease phosphorylation levels of JAK2 and Stat3 but were upregulated in FAM134B inhibition cells (Fig. 5F).

### JAK/Stat3 signaling pathway is required for FAM134B induced radiation sensitive

**Fig. 6:**
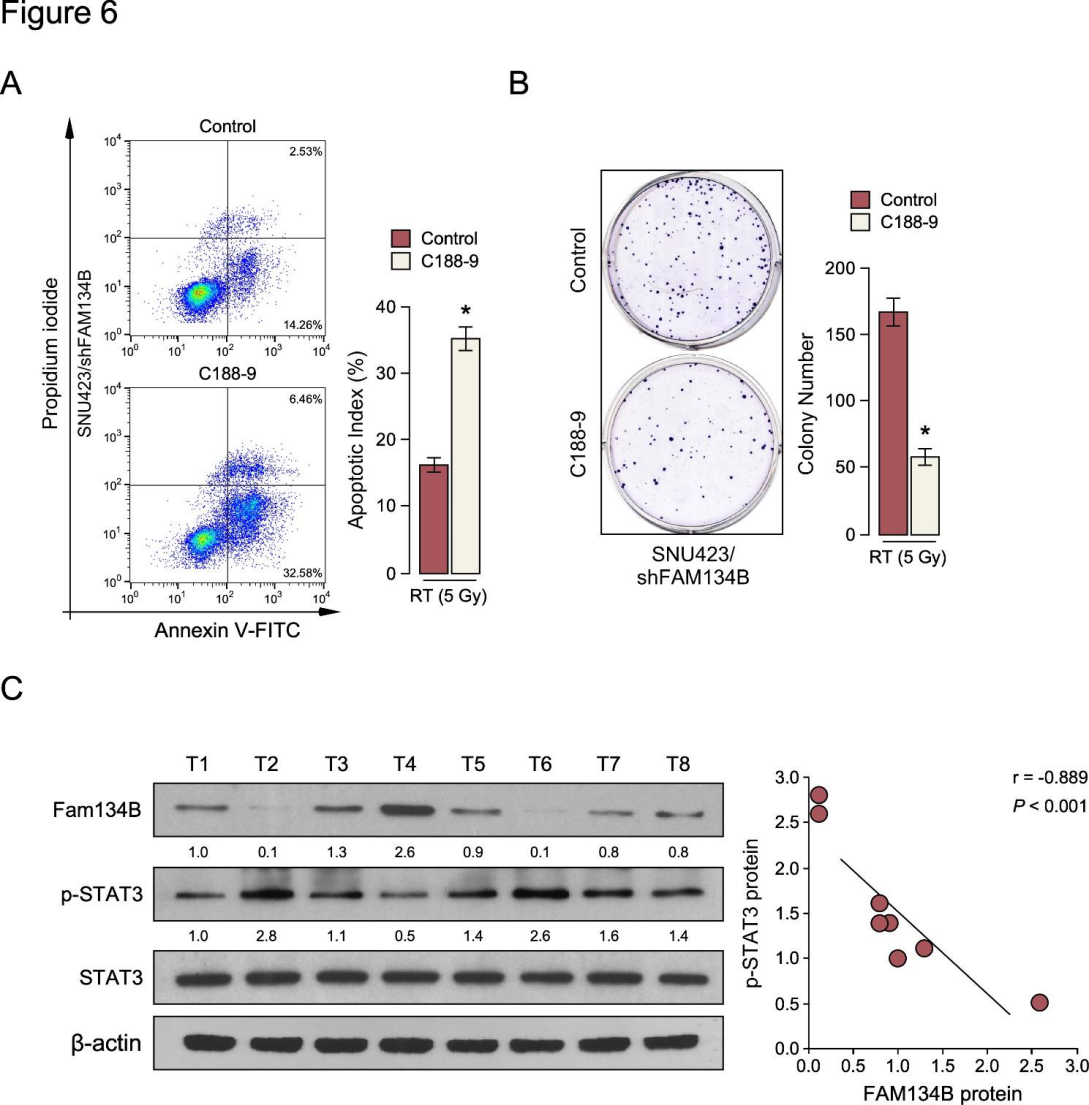
**JAK/Stat3 signaling pathway is required for FAM134B induced radiation sensitive** **A.** Annexin V-FITC and PI staining of the indicated cells. **B.** Colony formation analyze in the indicated cells. **C.** Correlation analysis of FAM134B expression and p-Stat3 expression in 8 freshly collected human HCC tissue samples (T); β-actin was used as a loading control. Each bar represents the mean ± SD of three independent experiments. * *P* < 0.05

Next, we investigated whether FAM134B mediated HCC radiation sensitive through JAK/Stat3 signaling pathway activation. Strikingly, Annexin V and colony formation assay shown that blockade of the JAK/Stat3 pathway by using C188-9, a specific inhibitor of Stat3 significantly decreased the effect of FAM134B on HCC radiation-sensitive (Fig. 6A-B). Moreover, FAM134B expression in 8 clinical HCC samples also conversely correlated with the protein levels of p-stat3 (r = -0.889, P < 0.01) (Fig. 6C). The above data supported the topics that downregulation of FAM134B in HCC JAK/Stat3 signaling pathway activation and confers radiation-resistance in HCC.

## Discussion

For a long time, the treatment of liver cancer mainly focused on the tumor in situ and ignored the treatment of intrahepatic metastasis, resulting in a very high recurrence rate [22]. It has been reported that the proportion of multicentric occurrence and intrahepatic metastasis of liver cancer is as high as 19.5% ~ 27.5% and 59.4% ~ 69.5% respectively [6, 23]. These intrahepatic micrometastasis lesions (≤ 0.5 cm) existed at the first diagnosis, but they could not be detected by current imaging techniques (including B-ultrasound, CT, Mr or PET / CT, etc.), which led to the fact that surgical resection and radical treatment were not “radical”. Among 2116 patients with liver cancer who were treated with interventional therapy because they were not suitable for surgery, more than 90% found cancer cell residues after interventional therapy, which eventually led to tumor recurrence and metastasis. Although the multicentric occurrence and intrahepatic metastasis of liver cancer are great obstacles to the above treatment methods, they are not suitable for radiotherapy [24, 25]. Therefore, radiosensitization of liver cancer is the main research direction at present. Herein, we provide evidence that FAM134B is downregulation and correlates with radiation sensitive in HCC. Overexpression of FAM134B confers radiation sensitive to HCC and actives the JAK2/Stat3 signaling pathway. Moreover, FAM134B overexpression decreased radiation-resistant, but FAM134B silencing restored the radiation sensitivity of HCC cells. These findings identify FAM134B/JAK2/Stat3 axis may be a potential target for overcoming radiation resistance in patients with HCC.

FAM134B has been found to be downregulated in multiple human cancers, and overexpression of FAM134B contributes to inhibit cancer growth both *in-vitro* and *in-vivo* via different mechanisms [26–29]. However, the expression of FAM134B has also been shown to be upregulated in esophageal squamous cell carcinoma compared to non-neoplastic tissues, and upregulation of FAM134B in ESCC induced significant cell proliferation and colony formation, and induce wound healing, migration, and invasion capacities of ESCC [14, 15]. These findings indicate that FAM134B functions as both an oncomir and tumor-suppressive miRNA depending on the tumor type. To investigate the clinical significance, biological function and the precise mechanism of action of FAM134B in HCC pathogenesis, we examined the FAM134B expression in HCC and found that FAM134B is downregulated in HCC, and FAM134B expression inversely correlated with the clinicopathological features and overall survival (p = 0.026), relapse-free survival(p = 0.0029), progression-free survival(p = 0.0089) and disease-specific survival(p = 0.015) of HCC patients, suggesting that FAM134B may be associated with the progression of HCC. Consistently, we provide evidence that downregulation of FAM134B confers radiation resistance to HCC and actives the JAK2/Stat3 signaling pathway. FAM134B overexpression decreased radiation-resistant, but FAM134B silencing restored the radiation sensitivity of HCC cells. Moreover, we found that downregulation of FAM134B enhanced radiation resistance by activating the JAK2/Stat3 signaling pathway. These results further support the notion that a single protein may have distinct functions in different cell types.

The Janus kinase 2/signal transducer and activator of transcription 3 (JAK2/STAT3) signaling pathway, a well-conserved and basic intracellular signaling cascade, is one of the most frequent molecular events in various cancers and activation of this pathway is thought to be an early event in tumorigenesis [30–33]. In the current study, by performing the public data analysis and combined with our experimental results, it is shown that FAM134B overexpression significantly repressed JAK2/STAT3 activity but inhibiting of FAM134B significantly increase JAK2/STAT3 activity in HCC. The above studies suggested that FAM134B may represent an important target for clinical intervention in HCC by controlling JAK2/STAT3 signaling, which gives hope for the further development of this compound as a drug used in HCC clinical oncology.

## Conclusions

In conclusion, for the first time we provide evidence that downregulation of FAM134B confers radiation resistance to HCC and actives the JAK2/Stat3 signaling pathway. FAM134B was found to be significant decreased in radiation-resistant HCC tissues and FAM134B overexpression decreased radiation-resistant, but FAM134B silencing restored the radiation sensitivity of HCC cells. Moreover, we found that downregulation of FAM134B enhanced radiation resistance by activating the JAK2/Stat3 signaling pathway. These findings identify FAM134B/JAK2/Stat3 axis may be a potential target for overcoming radiation resistance in patients with HCC.

## Electronic supplementary material

Below is the link to the electronic supplementary material.

Supplementary Material 1

Supplementary Material 2 Supplementary Material 2

Supplementary Material 3

## Data Availability

The datasets used and/or analyzed during the current study are available from the corresponding author upon reasonable request.

## Abbreviations

HCC: Hepatocellular Carcinoma
FLT3: FMS related receptor tyrosine kinase 3
FAM134B: Family with sequence similarity 134 member B
SD: Stable disease
PD: Progressive disease
CR: Complete response
PR: Partial response
IP/MS: Immunoprecipitation/mass spectrometry
co-IP: Co-immunoprecipitation
GSEA: Gene set enrichment analysis
ABCG2: ATP binding cassette transporter protein G2
JAK2/STAT3: Janus kinase 2/signal transducer and activator of transcription 3
SI: Staining index
