# Supplementary figures and images for: Elevated FAM134B expression induces radiation-sensitive in hepatocellular carcinoma
Source: BMC Cancer. 2023 Jul 17;23:671. doi: 10.1186/s12885-023-11030-x (PMC10353116; doi:10.1186/s12885-023-11030-x)

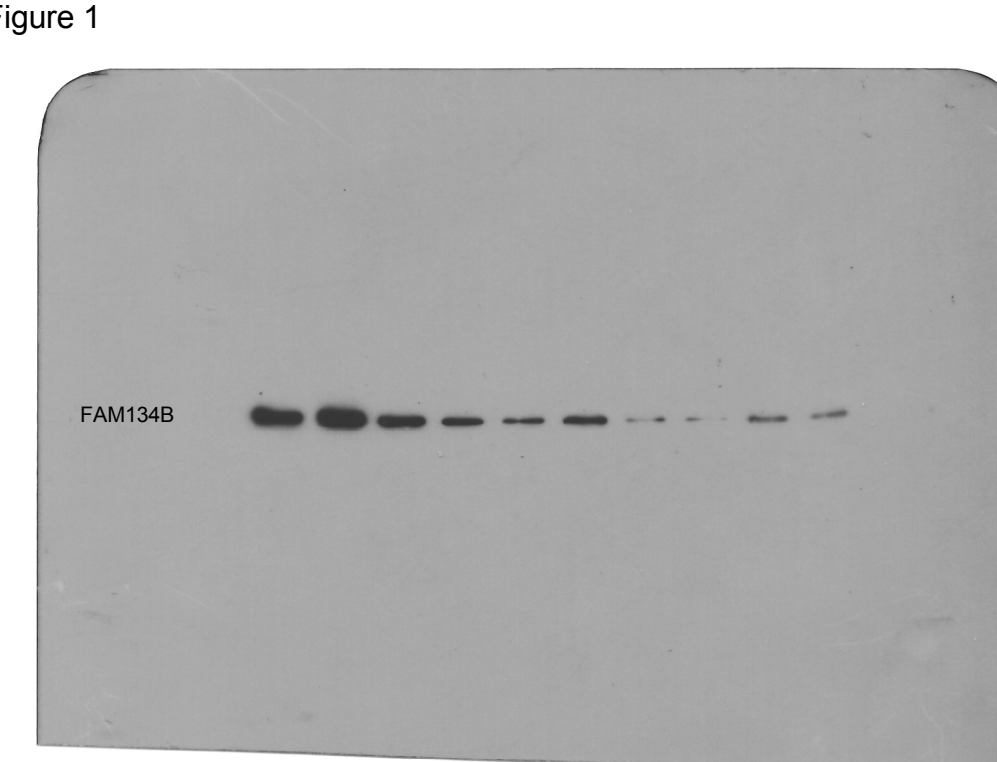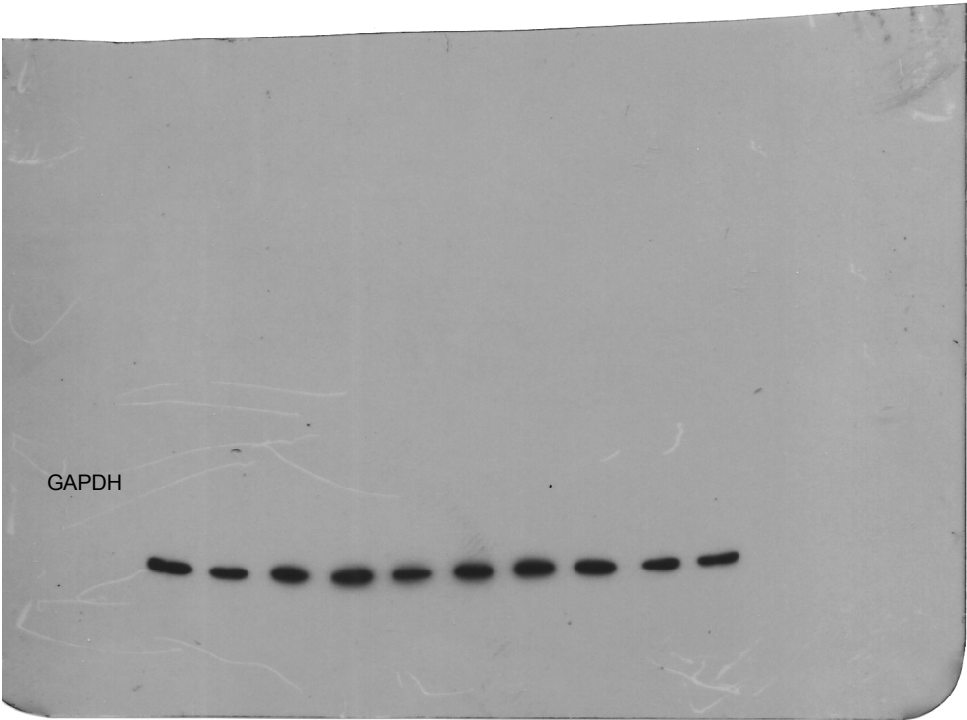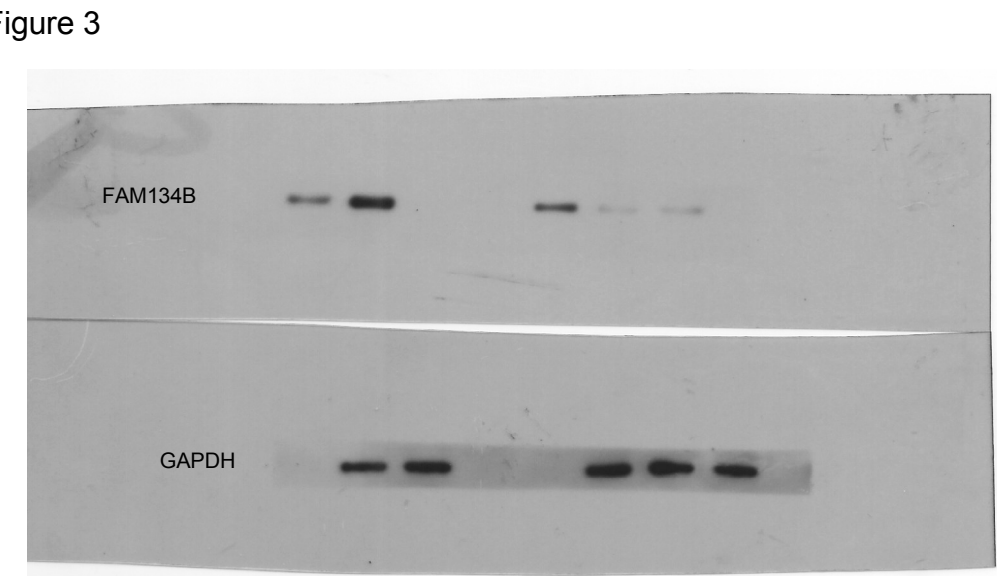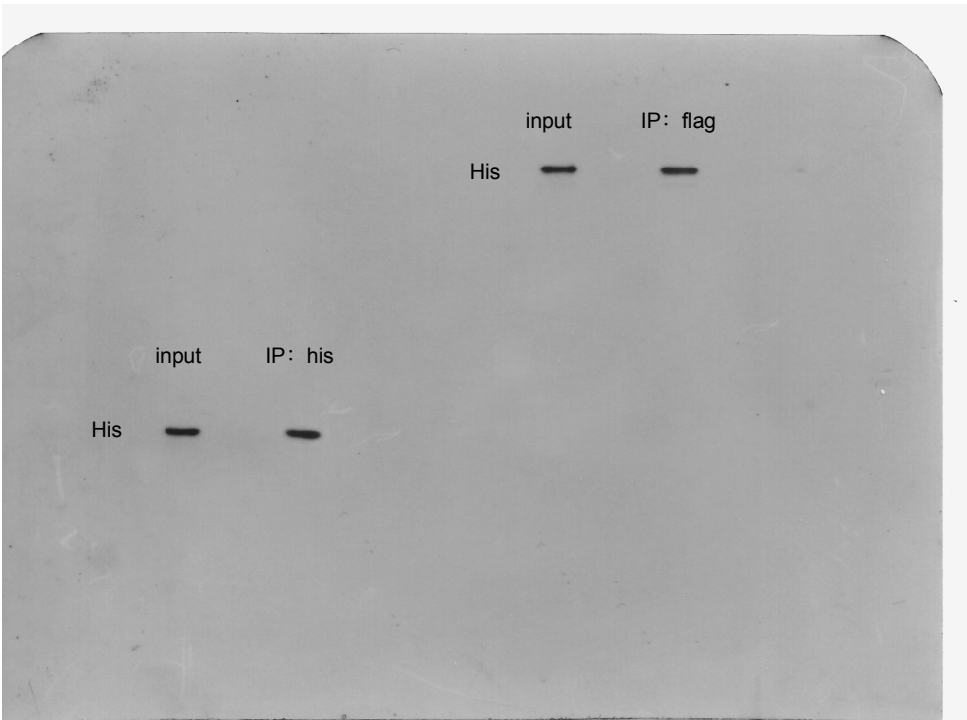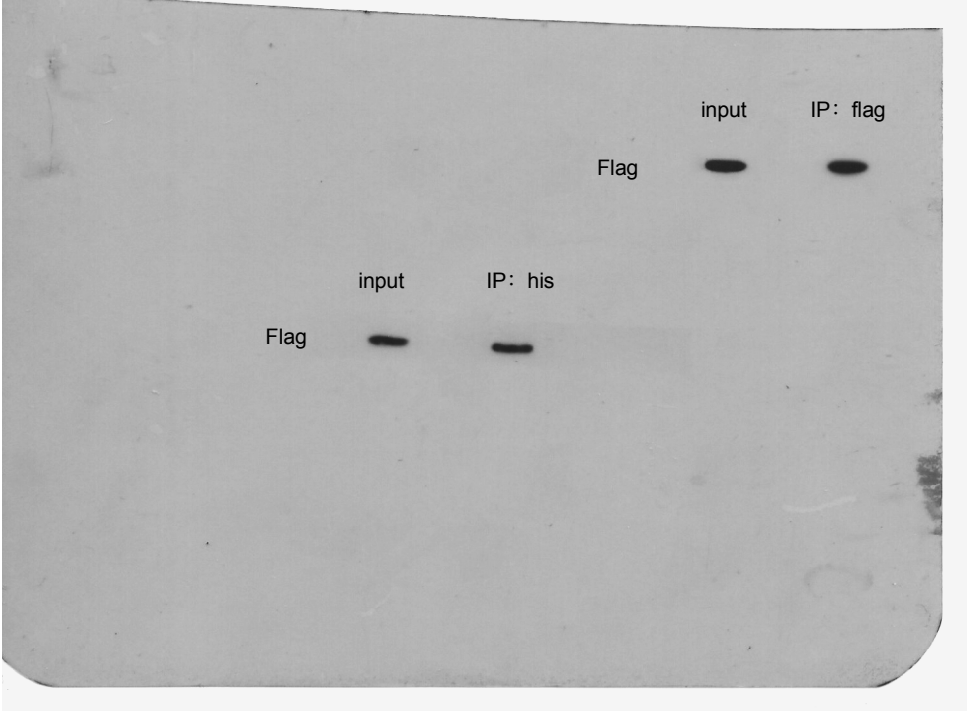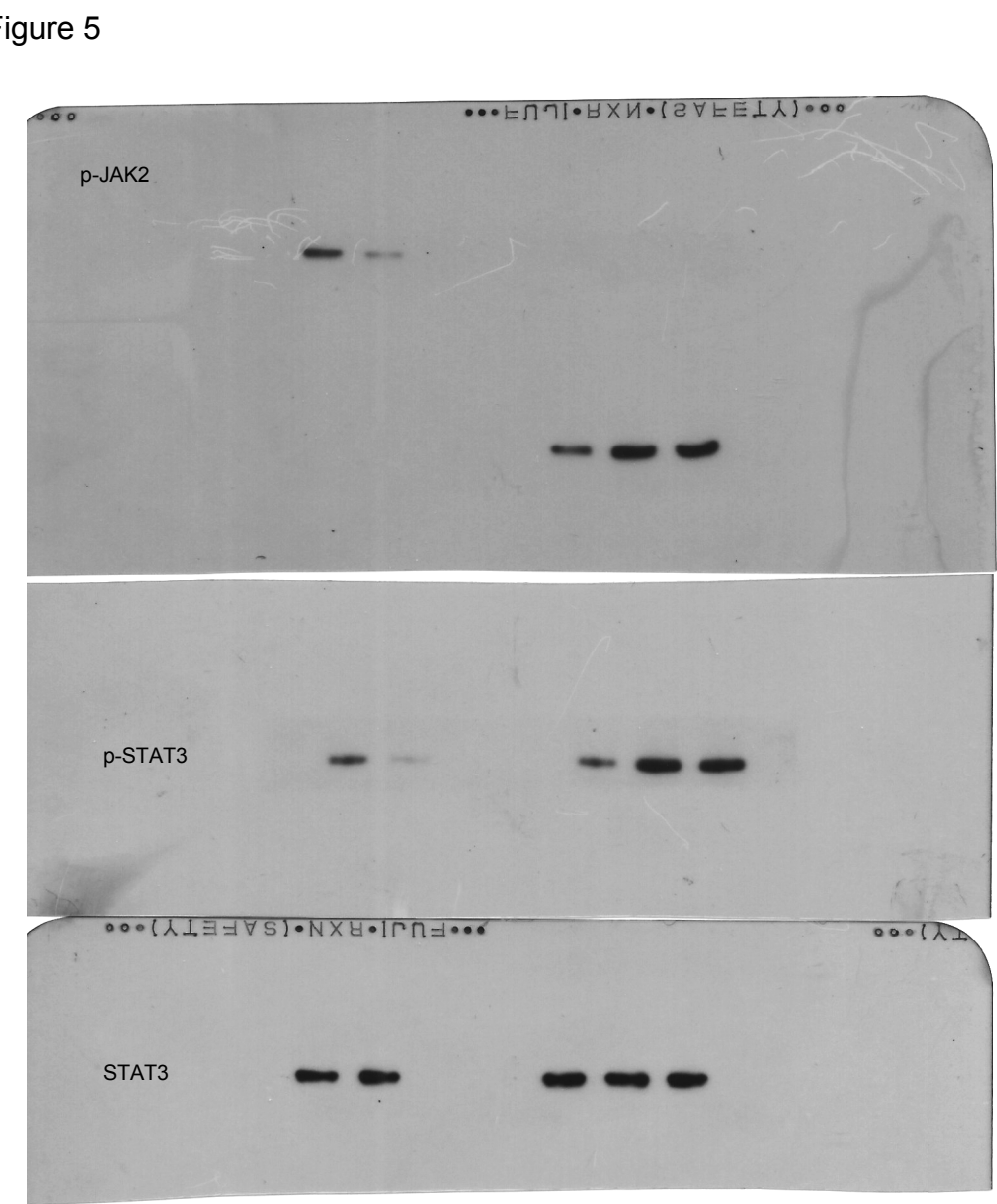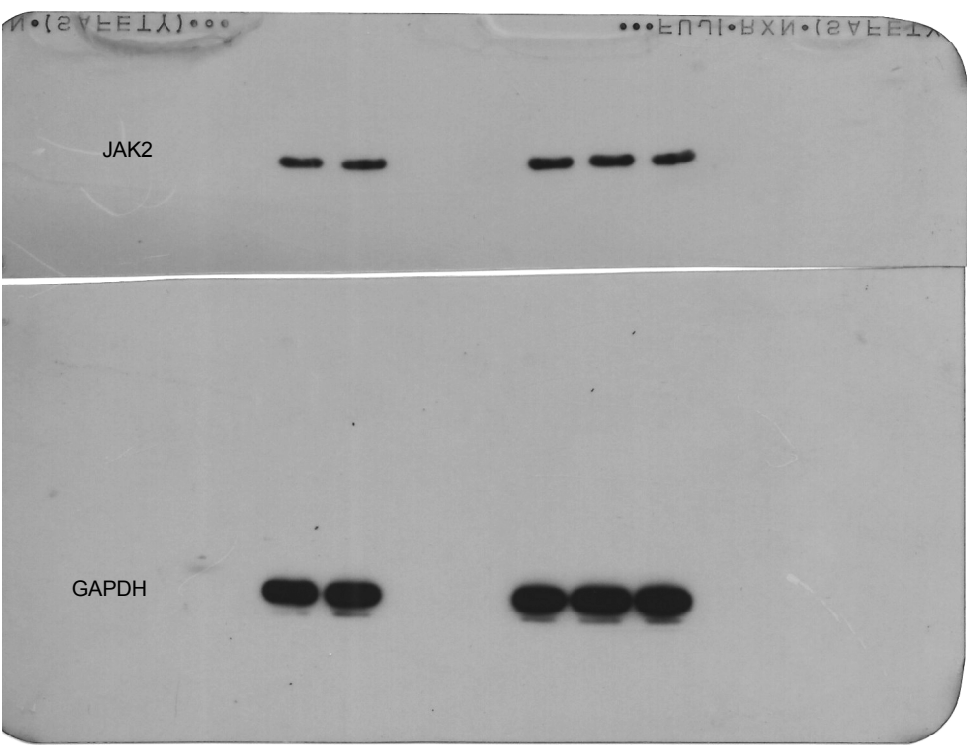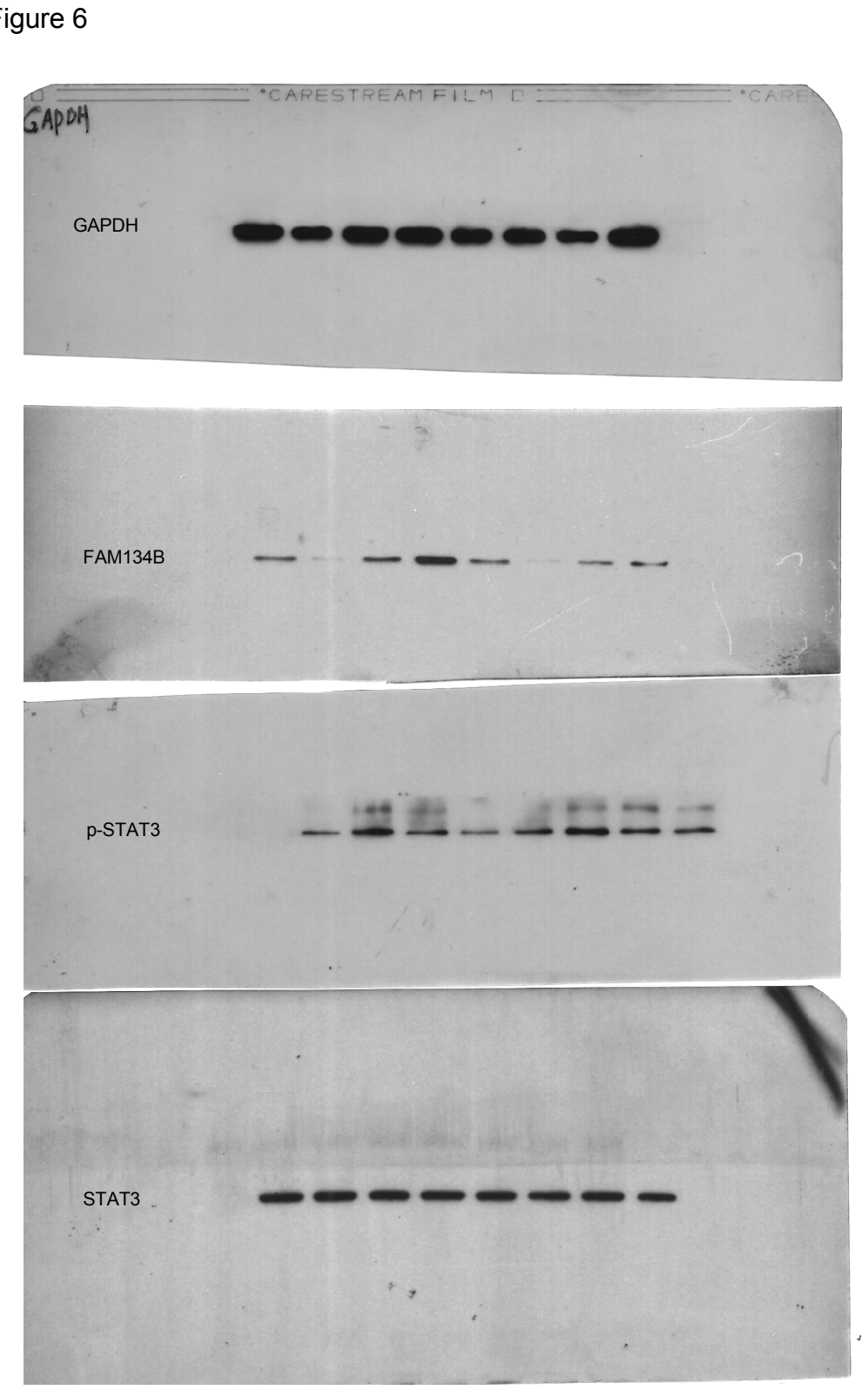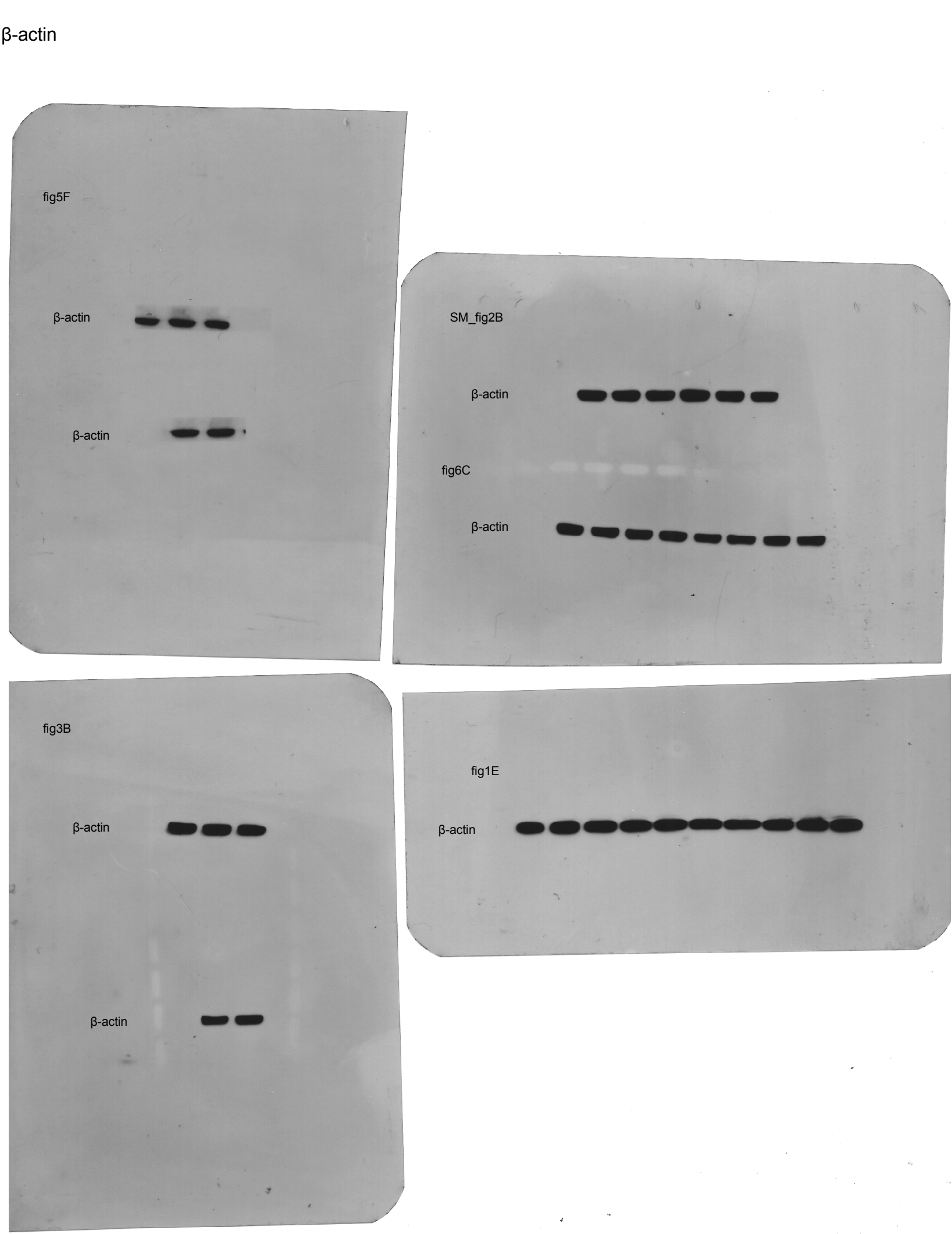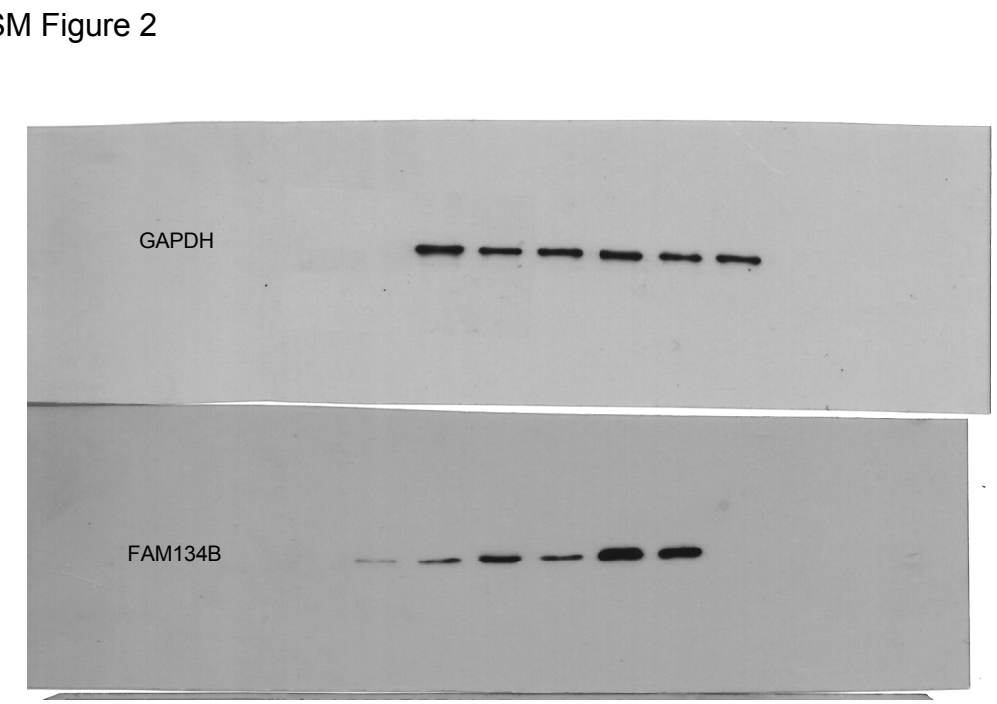

Supplement: Supplementary file 1 — Supplementary Material 1 [file 12885_2023_11030_MOESM1_ESM.pdf]

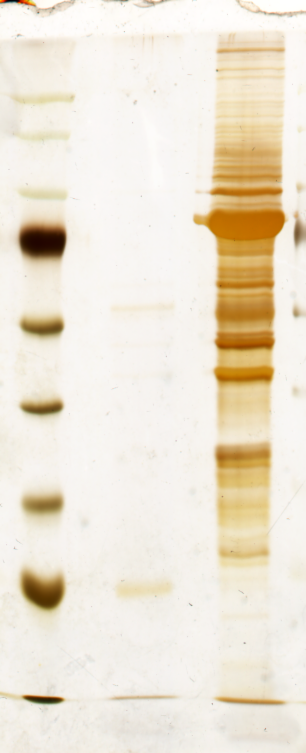

Supplement: Supplementary file 2 — Supplementary Material 2 [file 12885_2023_11030_MOESM2_ESM.png]
