# Supplementary material for: Elevated FAM134B expression induces radiation-sensitive in hepatocellular carcinoma
Source: BMC Cancer. 2023 Jul 17;23:671. doi: 10.1186/s12885-023-11030-x (PMC10353116; doi:10.1186/s12885-023-11030-x)
Supplement: Supplementary file 3 — Supplementary Material 3 [file 12885_2023_11030_MOESM3_ESM.pdf]

Supplemental

SM Figure 1

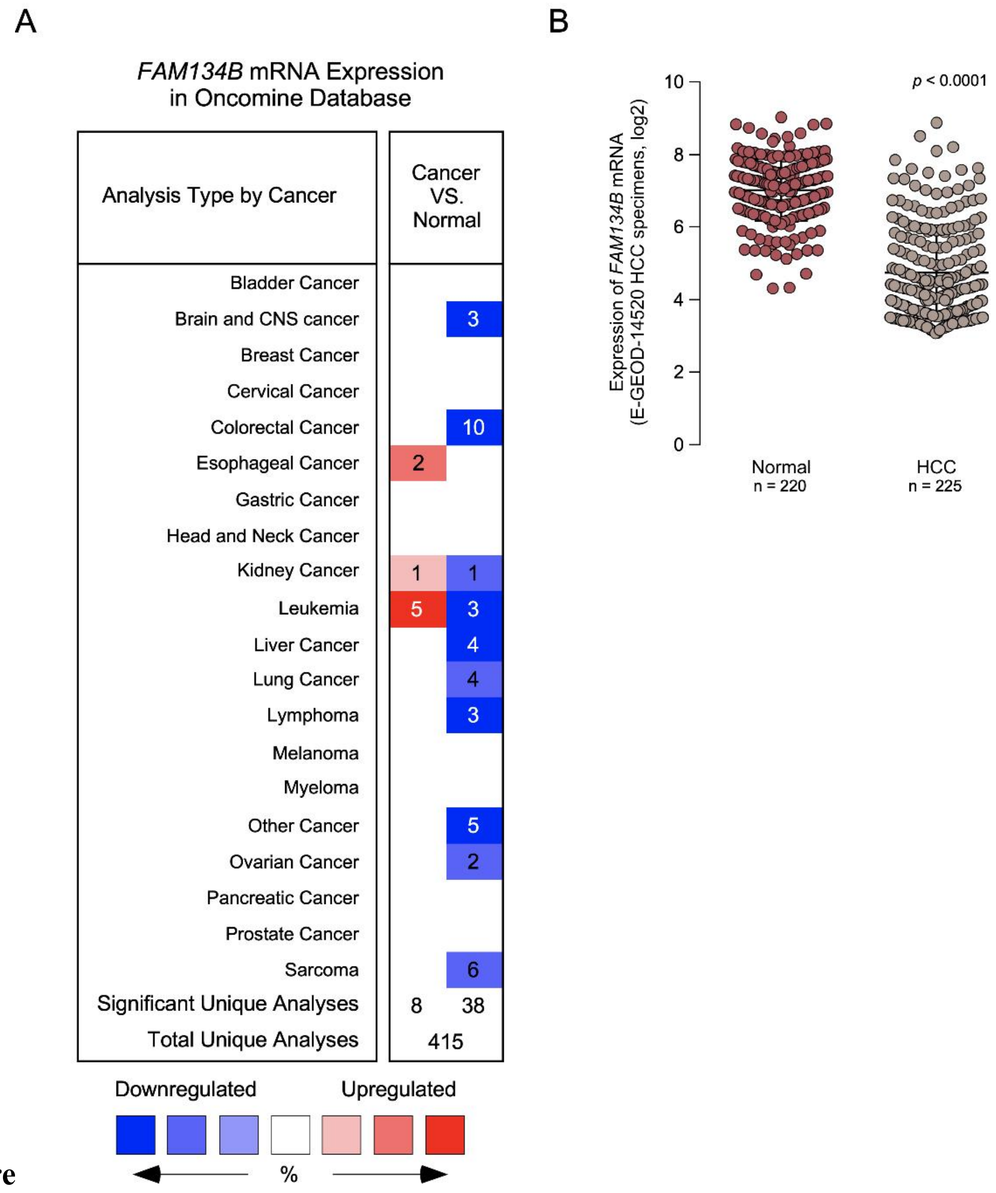

figure

Supplemental Figure 1 A. mRNA expression of FAM134B in Oncomine database in multiple cancers. **B.** Downregulated FAM134B in primary HCC tissues (Tumor) compared with normal tissue (Normal) (n = 445, E-GEOD-14520).

SM Figure 2

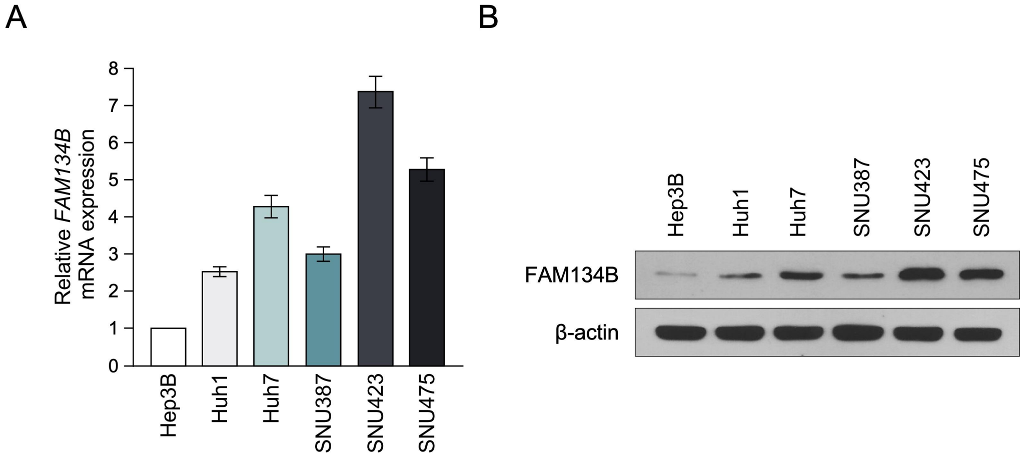

Supplemental Figure 2 A. Real-time PCR analysis of FAM134B expression in 6 HCC cell lines. **B.** Western blotting analysis of FAM134B expression in 6 HCC cell lines. β–actin was used as a loading control. Each bar represents the mean ± SD of three independent experiments.
